# History of Childhood Maltreatment and College Academic Outcomes: Indirect Effects of Hot Execution Function

**DOI:** 10.3389/fpsyg.2017.01091

**Published:** 2017-07-05

**Authors:** Marilyn C. Welsh, Eric Peterson, Molly M. Jameson

**Affiliations:** School of Psychological Sciences, University of Northern Colorado, GreeleyCO, United States

**Keywords:** child maltreatment, executive functions, academic achievement, academic adaptation, college students

## Abstract

College students who report a history of childhood maltreatment may be at risk for poor outcomes. In the current study, we conducted an exploratory analysis to examine potential models that statistically mediate associations between aspects of maltreatment and aspects of academic outcome, with a particular focus on executive functions (EF). Consistent with contemporary EF research, we distinguished between relatively “cool” EF tasks (i.e., performed in a context relatively free of emotional or motivational valence) and “hot” EF tasks that emphasize performance under more emotionally arousing conditions. Sixty-one male and female college undergraduates self-reported childhood maltreatment history (emotional abuse and neglect, physical abuse and neglect, and sexual abuse) on the Childhood Trauma Questionnaire (CTQ), and were given two EF measures: (1) Go-No-Go (GNG) test that included a Color Condition (cool); Neutral Face Condition (warm); and Emotion Face condition (hot), and (2) Iowa Gambling Task (IGT), a measure of risky decision making that reflects hot EF. Academic outcomes were: (1) grade point average (GPA: first-semester, cumulative, and semester concurrent with testing), and (2) Student Adaptation to College Questionnaire (SACQ). Correlational patterns suggested two EF scores as potential mediators: GNG reaction time (RT) in the Neutral Face condition, and IGT Block 2 adaptive responding. Indirect effects analyses indicated that IGT Block 2 adaptive responding has an indirect effect on the relationship between CTQ Total score and 1st semester GPA, and between CTQ Emotional Abuse and concurrent GPA. Regarding college adaptation, we identified a consistent indirect effect of GNG Neutral Face RT on the relationship between CTQ Emotional Neglect and SACQ total, academic, social, and personal–emotional adaption scores. Our results demonstrate that higher scores on a child maltreatment history self-report negatively predict college academic outcomes as assessed by GPA and by self-reported adaptation. Further, relatively “hot” EF task performance on the IGT and GNG tasks serves as a link between child maltreatment experiences and college achievement and adaptation, suggesting that hot EF skills may be a fruitful direction for future intervention efforts to improve academic outcomes for this population.

## History of Child Maltreatment and College Academic Outcomes: Mediation by Execution Function

College students with a developmental history of child maltreatment represent a significant subset of the university population. We believe these students may comprise an important but overlooked group for targeted interventions aimed at promoting educational success. Although we are not aware of any definitive epidemiological studies, current research suggests that the base rate of maltreatment history among university students ranges from the mid-20% to more than 40% (e.g., Duncan, 2000; Freyd et al., 2001; Gibb et al., 2009). Across several semesters of research in our laboratory, recruiting from a mid-sized university in the western United States, we have consistently yielded a base rate of approximately 30% students with a maltreatment history. Presumably, differences in base rate across the extant studies reflect characteristics related to each individual college setting (the average socioeconomic status of students, etc.) or factors specific to mechanism of recruitment (specific trauma measures employed, sampling methods, etc.). Moreover, all studies of childhood history of maltreatment rely on self-report or clinical interview measures (Roy and Perry, 2004), which results in an unavoidably heterogeneous population of students with regard to the timing, nature, and severity of childhood trauma. Nevertheless, college students reporting childhood maltreatment represent an important, understudied subgroup of the general student population that are at increased risk for a range of cognitive, emotional, behavioral, and psychiatric sequelae (e.g., Mersky and Topitzes, 2010).

Whereas the negative impact of child maltreatment on academic performance in children has been well documented (e.g., Perzow et al., 2013; Kiesel et al., 2016), an examination of college achievement and adaptation in students with a history of maltreatment has not been a priority in research. In the only longitudinal study to date, Duncan (2000) followed 210 college freshmen, 36% who were identified as having experienced child abuse (emotional, physical, and/or sexual). Four years later, those students reporting multiple forms of abuse or sexual abuse only were significantly less likely to be still enrolled in college than non-victims, and PTSD symptoms during the second week of freshman year interacted with abuse to predict attrition. A small empirical literature suggests that college students reporting a history of childhood trauma also report lower levels of college adaptation and adjustment (Banyard and Cantor, 2004; Elliot et al., 2009; Maples et al., 2014). In a rare study of grade point average (GPA) as a college outcome, Jordan et al. (2014) reported that women who had been sexually assaulted as adolescents not only entered college with lower high school GPAs, but also earned lower GPAs by the end of their first year in college. This emerging literature suggests adverse college adaptation and achievement in students reporting a history of maltreatment; however, very little is known about which specific maltreatment sequelae mediate poor adaptation to the college environment. Identification of such variables would provide potential directions for effective intervention to enhance the chances of academic success and its resultant health and economic benefits (e.g., Leonhardt, 2014; Pew Social Research Center, 2014).

While it is clear that maltreatment history confers risk for poor outcome, the problem of heterogeneity among the maltreatment group presents an inherent challenge for the identification of individuals who may carry the greatest risk. This reflects the potential range of different negative experiences that may have influenced retrospective self-report, but it also reflects the varying degrees of individual resilience. Given the multifactorial relationship between developmental history and outcome, we examine current phenotype in an effort to determine which individuals who report maltreatment may be at the greatest risk for poor outcome. This study represents the first in the published literature to examine a novel potential mediator in the pathway between a self-reported history of maltreatment in childhood and academic achievement and adaptation in college: the indirect effects of executive function (EF) processes, and more specifically “hot” EFs.

Executive function (including planning, working memory, inhibition, and flexibility) is a particularly relevant cognitive domain to examine for three reasons. First, evidence shows that stress early in life has deleterious effects on the development and function of the prefrontal cortex (e.g., McEwen, 2008), the brain system that mediates EF (e.g., Kane and Engle, 2002). Performance in traditional cool EF has been particularly associated with the dorsolateral prefrontal cortex. However, since the seminal work of Bechara et al. (2000) and Bechara (2004), researchers have examined the role of orbitofrontal and ventromedial regions of prefrontal cortex in contexts that involve managing heightened emotional arousal (Goel and Dolan, 2003). Given our interest in hot executive processes, the evidence of functional (Etkin and Wager, 2007) and structural (Gold et al., 2016) imaging of ventromedial impairment associated with trauma is particularly relevant. Consistent with these early neurocognitive impacts, deficits in EF processes mediated by the prefrontal cortex have been demonstrated in adolescents, college students, and adults with self-reported maltreatment histories (Spann et al., 2012; Nikulina and Widom, 2013; Kirk-Smith et al., 2014; Mothes et al., 2015; Vasilevski and Tucker, 2016). For example, college women reporting a history of repeated childhood sexual abuse exhibited performance deficits on a modified Go-No-Go task (Navalta et al., 2006), one of the measures used in the current study.

Second, the types of self-regulatory behaviors that are subsumed within the construct of EF have been linked empirically to success in school for children (e.g., St Clair-Thompson and Gathercole, 2006; Masten et al., 2012; Willoughby et al., 2016) and have recently been the target of interventions to improve academic performance (Diamond and Lee, 2011; Diamond, 2012). Although there are established links between individual differences in EF and academic achievement and adjustment for children, parallel research with the college population is relatively sparse. Individual differences in EF, such as attentional control, planning, self-monitoring and self-regulation have been found to predict college achievement in terms of credits achieved (Baars et al., 2015) and academic task completion (Rabin et al., 2011; Gustavson et al., 2015). Thus, deficits in EF related to childhood maltreatment and the link between EF and college success, clearly point to examining EF skills as a potential mediator in this novel investigation of college students.

Finally, we have suggested (Peterson and Welsh, 2014; Welsh and Peterson, 2014) that the examination of EF in real-world settings such as the college environment would benefit from the use of tasks that are specifically designed to measure these skills in more arousing contexts, referred to as hot EF (Zelazo and Carlson, 2012; Peterson and Welsh, 2014; Welsh and Peterson, 2014). Evidence from the trauma literature makes clear that individuals with a maltreatment history are more likely to have particular difficulty with emotion regulation (e.g., Cloitre et al., 2005; Etkin and Wager, 2007), which could easily disrupt executive processes in real-world contexts. One promising approach to examining hot executive processes involves adapting a traditional executive instrument (e.g., Stroop Interference, Go-No-Go) to enable an examination of the potential role of “heating” (i.e., adding an emotional or motivational component), as we did in the current study. Such a task manipulation can allow for a comparison of task performance between relatively unheated (i.e., cool) and heated conditions. For the study of maltreatment history, task manipulations that involve replacing neutral stimuli with trauma-relevant, emotionally valenced stimuli may be particularly fruitful. Two research groups manipulated the Stroop task of inhibition and flexibility by introducing threat word stimuli (Fontenot et al., 2015) and emotion face stimuli (Caldwell et al., 2014), finding poorer performance by young adults reporting a history of maltreatment particularly in the heated conditions. Cromheeke et al. (2014) administered the Spatial Emotional Match to Sample task, a working memory test heated with the use of neutral and emotion faces, to adult women with differing trauma histories. Women with child or adult histories of sexual or physical abuse exhibited specific impairments on the more difficult task conditions and only with the happy emotion faces. In the current study, we heated the traditionally cool EF task, Go-No-Go, by including arousing face stimuli to investigate whether history of childhood maltreatment would specifically co-vary with performance in these conditions.

As reviewed above, the literature suggests that individuals with a child maltreatment history are at risk for both negative college outcomes and deficits in EF, and a specific focus on hot executive processes may be particularly relevant to the study of cognitive sequelae of maltreatment.

The college academic setting places demands on emotion regulation (in testing settings, interpersonal contexts, etc.) and it seems likely that hot EF tasks may be sensitive to deficits associated with emotion regulation in college students, as it is in children (e.g., Woltering et al., 2015). In summary, a small extant literature suggests that a history of childhood maltreatment confers risk for college outcomes; however, it remains a relatively understudied area of inquiry. Research demonstrates that the prediction of college academic achievement and adaptation is a multifactorial enterprise, such that any single variable will predict only a small amount of variance in these outcomes (e.g., more proximal variables such as motivation and metacognition predicting GPA yielded correlations ranging from 0.13–0.39; Komarraju and Nadler, 2013). Even a study that examined the degree to which intelligence predicted college GPA yielded correlations ranging from 0.15–0.21 (Murray and Wren, 2003). Not only is the examination of academic outcomes in this potentially vulnerable subgroup of college students relatively rare, we know of no published study of EF processes as a potential mediator of this association.

### Purpose and Research Questions

Our study addresses the question: which EFs have an indirect effect on the relationship between a self-reported history of exposure to different types of child maltreatment (emotional, physical, and sexual abuse, as well as neglect) and college GPA and adaptation? Recent studies have targeted a range of interesting adulthood outcomes of maltreatment, such as mental health, risk taking, social relationships, cognition, and academic performance (e.g., Duncan, 2000; Higgins and McCabe, 2000; Banyard and Cantor, 2004; Cromheeke et al., 2014). However, very few published studies have tested mediational models linking maltreatment history to a given outcome (e.g., Allwood and Bell, 2008; Bachrach and Read, 2012). We are not aware of any published studies that have addressed the central aim of the current exploratory, descriptive study: EF processes as mediators between experiences of child maltreatment and academic outcomes in college students.

## Materials and Methods

### Participants

The sample included 64 undergraduate students (17 males, 47 females; *M* = 19.33, *SD* = 2.21) who volunteered to participate through the Psychological Sciences participant pool. These participants volunteered for an earlier screening session with the study name “Stressful Life Experiences, Cognition, Emotion, and Academic Adaptation.” In this earlier session, we administered a maltreatment screen using the Child Trauma Questionnaire (see descriptions below), the Vocabulary subtest of the WAIS-IV, and a demographics survey that included years of maternal education (as a proxy for SES; see Zhang and Wang, 2004; Classen and Hokayem, 2005; Baum and Ruhmb, 2009). Screening participants also were given the opportunity to provide informed consent for us to contact them back for participation in a future study (i.e., the current study) and to allow us to monitor their academic progress longitudinally (i.e., recording academic outcomes such as grade point average, GPA, at the conclusion of each semester). The only exclusionary criteria applied in this invitation were: (1) not born in the United States (given potential differences in self-reporting child maltreatment history), (2) no consent given for follow-up, and (3) validity problems on the Childhood Trauma Questionnaire (see description, below). The descriptive statistics for age, gender, Vocabulary subtest raw score, and years of maternal education are displayed in **Table 1**.

**Table 1:** Descriptive statistics for variables used in subsequent analyses.

		Min.	Max.	Mean	*SD*
Demographic Variables					
	Age	18	34	19.33	2.21
	SES	8	18	14.73	2.25
	WAIS Vocabulary Score	5	16	11.37	2.45
Predictor Variables					
	CTQ Emotional Abuse	5	22	10.62	4.99
	CTQ Physical Abuse	5	22	7.56	4.16
	CTQ Sexual Abuse	5	25	6.49	3.47
	CTQ Emotional Neglect	5	25	9.79	4.72
	CTQ Physical Neglect	5	20	6.83	2.96
	CTQ Total	25	93	41.29	15.19
Mediating Variables					
	Neutral Face GNG RT	299.74	618.97	502.82	61.99
	Anger Face GNG RT	285.73	595.31	491.51	57.28
	Fear Face GNG RT	322.67	589.16	471.78	53.45
	Neutral Face GNG Accuracy	0.47	1	0.93	0.08
	Anger Face GNG Accuracy	0.44	1	0.90	0.11
	Fear Face GNG Accuracy	0.64	1	0.89	0.10
	IGT Block 1	-18	16	-2.71	6.05
	IGT Block 2	-10	20	4.29	7.10
	IGT Block 3	-12	20	5.90	7.64
	IGT Block 4	-10	20	6.55	8.41
	IGT Block 5	-20	20	4.74	9.94
Outcome Variables					
	SACQ Academic	76	192	138.76	28.26
	SACQ Social	53	172	125.41	23.47
	SACQ Personal/Emotion	28	119	77.75	21.19
	SACQ Attachment	50	125	91.9	17.94
	SACQ Total	236	538	402.35	70.01
	First semester GPA	0.00	4.00	2.50	1.06
	Spring 2016 GPA	0.20	4.00	2.81	0.86
	Current Cumulative GPA	0.10	3.98	2.68	0.86


*SACQ scales: Academic Adjustment; Social Adjustment; Personal/Emotional Adjustment; Attachment (to college); Total Adjustment. GNG Accuracy scores for the No Go conditions. IGT scores reflect number of adaptive choices – number of maladaptive choices.*

### Materials and Procedures

#### Childhood Trauma Checklist (CTQ)

The CTQ (Bernstein et al., 2003) is a retrospective self-report of childhood and adolescent abuse and neglect. The measure demonstrates adequate reliability for each scale (Bernstein et al., 1994), and has been validated against clinician’s reports of history of childhood maltreatment that included evidence from Child Protective Services and court records (Bernstein et al., 1997). Across 28 items, respondents rate the frequency of occurrence for each item, ranging from 1 (never true) to 5 (very often true). The CTQ yields five clinical scales, three of which assess abuse (Emotional, Physical, and Sexual) and two that assess neglect (Emotional and Physical). The total CTQ score was used as an overall child maltreatment score, while summed scores from each subscale were used to indicate degree of severity for each type of maltreatment. The CTQ includes three validity-check questions regarding the overall quality of family life during the participant’s childhood and a total score of 3 or 4 indicates a potential bias toward social desirability in the form of over-reporting high-quality childhood experiences. Therefore, participants who scored a 3 or 4 in the original screening sample were not invited to participate in the current study.

#### Go-No-Go (GNG)

This classic EF task assesses conflict monitoring and inhibitory control, and can be easily manipulated to include both “cool” stimuli (e.g., colors, shapes) and “hot” stimuli (faces) to examine the involvement of both dorsolateral and ventromedial prefrontal cortical regions (e.g., Hare et al., 2008). In each trial, participants see a target that varies (i.e., a blue shape versus a yellow shape). For one stimulus (the “Go” condition) participants are instructed to make a reaction time (RT) button press response; for the other stimulus (the “No-Go” condition), participants must withhold a response. We adapted the traditional Go-No-Go paradigm to include three blocks: one cool (stimuli were colors, red versus blue); one somewhat heated (stimuli were neutral faces, male versus female); and a third, hot (emotion faces, male and female faces displaying anger or fear). All participants completed the blocks in the following order: Color, Neutral Face, Emotion Face. For each block, there were 15 practice trials and 120 test trials with 66.6% Go trials in the Color block and 75% Go trials in the Neutral Face and Emotion Face blocks (Casey et al., 2011). The stimulus was presented for 1 s (unless terminated earlier by a participant response) and the inter-stimulus interval was 1 s. Go versus No-Go stimuli were counterbalanced across participants such that in the Color block, half of the participants made a reaction time response to the blue circle (i.e., go trials), and the other half responded to the yellow circle as the Go stimulus. In both the Neutral and Emotion Face blocks, half of the participants responded to the male face and half to the female face, with the constraint that the gender of the Go stimulus be equal across both male and female participants. For the Emotion Face block, half of the Go stimulus faces displayed an angry emotion and half displayed a fearful emotion. The source of the face stimuli was the NimStim Face Stimulus Set^1^. Participants were instructed to respond as quickly as possible to the designated Go stimulus while maintaining reasonable accuracy and to withhold their response to the No-Go stimulus. The dependent measures were RT to the Go stimuli and accuracy of responding (percentage correct) to both the Go stimuli and the No-Go stimuli in each of the three blocks of trials. Within the Emotion Faces block, RT and accuracy were examined separately for the angry and fear faces.

#### Iowa Gambling Task (IGT)

This published, standardized, computerized task (Chan et al., 2008) represents a simulated card game to examine risky decision making and learning from feedback. It has been cited in the literature as a hot EF task (e.g., Zelazo and Carlson, 2012) and performance has been associated with the orbitofrontal/ventromedial region of the prefrontal cortex (Bechara et al., 1994; Damasio, 1999). In their review of the IGT, Buelow and Suhr (2009) conclude that the task is a valid instrument for identifying decision making deficits (i.e., risky decision making) in a range of at-risk and clinical populations, such as substance abusers, pathological gamblers, Obsessive-Compulsive Disorder, Schizophrenia, and Attention Deficit Hyperactivity Disorder. The task presents the participant with 100 trials in which he or she selects a card from one of four decks and each selection is followed by a hypothetical monetary gain or loss, or both. Two of the decks (A and B) yield an initial rapid gain followed by loss across the decks while the other two decks (C and D) confer slow gains resulting in an overall positive outcome. Therefore, the IGT includes 100 selections from four decks, determined by the participant. The scoring typically divides the 100 selections in five blocks of 20 selections each, which differ across participants depending on their deck selections. The score indicates the number of adaptive (less risky) choices minus the number of maladaptive (more risky) choices for each of five blocks of 20 trials. A positive score reflects relatively more adaptive choices on that block. Participants began the task with a hypothetical $2000 such that they could finish above or below this level. To further heat the task to elicit hot EF, participants were told that they if they finished the task with more than $2000 (i.e., a positive outcome) they would receive a state lottery $1 scratch ticket.

#### Student Adaptation to College Questionnaire (SACQ)

This normed and standardized, 67-item, Likert-scale self-report instrument (Baker and Siryk, 1999) assesses overall adjustment to college (total score), as well as adjustment in four specific areas: Academic Adjustment, Personal–Emotional Adjustment, Social Adjustment, Attachment (to the institution). The survey is administered in 15–20 min and has been successfully used to identify college students who are at risk for attrition (Credé and Niehorster, 2012). Norms are based on a sample of more than 1,300 male and female college freshmen and stratified by semester of attendance (first and second semesters in college).

#### Grade Point Average (GPA)

Three GPA indices were taken directly from the participants’ official academic transcripts: (1) GPA earned in their first semester at the university; (2) GPA earned during the semester in which the testing took place; and (3) Cumulative GPA across all semesters at the university.

### Overall Procedure

All participants were recruited from a larger Screening Session (*N* = 120). Participants who scored in the moderate to severe range in any one of the five CTQ subscales (approximately 33% of the screening sample) and participants scoring at lower levels on CTQ subscales were invited back participate in a lab visit, which included the IGT, GNG, and other tasks and questionnaires, as part of a larger study. Of the 80 participants targeted for the lab visit, 16 did not participate in the lab visit because they could not be contacted, could not be scheduled, declined to participate, or did not show up for the scheduled session. The CTQ was individually administered during a single test session of 1 h. The GNG, IGT, and SACQ were administered in individual lab visits approximately 2 h in length. The GPA information was retrieved from the university academic records system approximately 2 weeks after the end of the semester.

### Data Analysis

To answer the main questions about the indirect effects of potential mediating EF variables on the relationship between self-reported history of child maltreatment and college outcomes, indirect effects analysis using bias-corrected bootstrapping via Hayes (2013) PROCESS macro for SPSS was utilized; this procedure analyzes the confidence intervals to determine indirect effects of mediating variables. As Preacher and Selig (2012) state, “bias-corrected bootstrapped confidence intervals have fairly accurate Type I error rates and higher power when compared to competing methods” (p. 81), and other researchers have demonstrated that bias-corrected bootstrapping procedures require the smallest sample size of several methods of analyzing indirect effects to achieve comparable levels of power (Fritz and MacKinnon, 2007). While others have found increases in Type I error rates with bias-corrected bootstrapping in small sample sizes (e.g., Fritz and MacKinnon, 2007; Fritz et al., 2012), this statistical approach is still considered to be the strongest and most robust method of analyzing indirect effects. Hayes (2009) referred to it as one of the more valid and powerful methods for testing intervening variable effects. In fact, Fritz and MacKinnon (2007) provide the recommendation that researchers use the bias-corrected bootstrap test for mediation analyses because of its increased power. Further, to determine which EF variables might have an indirect effect on these relationships, correlation analyses were conducted between predictor, potential mediating, and outcome variables, and the magnitude of the relationships was examined for potential mediators. While we also considered significance of the relationships, the magnitude of the correlations was of more import because of the exploratory and novel nature of this research.

## Results

### Descriptive Statistics

The means and standard deviations of all variables included in subsequent analyses are included in **Table 1** below. While demographic variables likely influence the relationships between history of child maltreatment and college outcomes, the relatively small sample size and exploratory nature of this study resulted in a decision to not statistically control for these variables.

### Associations between Childhood Trauma, Academic Outcomes, and Executive Functioning Performance

Because the nature of this research is exploratory, correlational analyses were conducted to guide our decision making on variables with potential indirect effects. This is considered to be steps one and two (i.e., show that the predictor and outcome are correlated; show that the predictor and mediators are correlated) of conducting traditional mediational analyses (see, for example, Baron and Kenny, 1986). Based on the results of these analyses, five potential mediators were found: GNG Neutral Face RT, GNG Fear Face RT, GNG Anger Face RT, IGT Block 2 adaptive reasoning, and IGT Block 3 adaptive reasoning. Mediators explored in the indirect analyses satisfied the requirement of associations with both the predictor (CTQ) and outcome (academic) variables.

Potential mediator, IGT Block 2 adaptive responding, correlated significantly with CTQ Emotional Abuse, Emotional Neglect, and CTQ Total Score (*r* = -0.28, *r* = -0.26, *r* = -0.28, respectively). Moreover, IGT Block 2 adaptive responding correlated significantly with cumulative GPA, concurrent GPA, and first semester GPA (*r* = 0.30; *r* = 0.36, *r* = 0.27, respectively). IGT Block 3 adaptive responding correlated significantly with CTQ Emotional Abuse (*r* = -0.26), as well as with cumulative GPA and first semester GPA (*r* = 0.29; *r* = 0.30).

Other potential mediators were identified in the GNG task. GNG Neutral Face RT correlated with CTQ Emotional Abuse (*r* = -0.23), CTQ Emotional Neglect (*r* = -0.23), and CTQ Total (*r* = -0.22), and GNG Fear RT correlated with CTQ Emotional Abuse (*r* = -0.29). Additionally, GNG Neutral Face RT correlated significantly with SACQ Academic Adjustment, Personal Emotional Adjustment, Attachment, and Total Score (*r* = 0.34; *r* = 0.35, *r* = 0.26; *r* = 0.39), as well as with all three GPA measures (*r* = 0.32 with cumulative, *r* = 0.29 with concurrent, and *r* = 0.32 with first semester). GNG Fear Face RT correlated significantly with SACQ Personal Emotional Adjustment (*r* = 0.25) as well as concurrent GPA (*r* = 0.25). GNG Anger Face RT correlated significantly with CTQ Emotional Abuse (*r* = -0.29), CTQ Total (*r* = -0.25), SACQ Personal Emotional Adjustment (*r* = 0.29), and SACQ Total (*r* = 0.26).

### Indirect Effects Analysis

**FIGURE 1:**
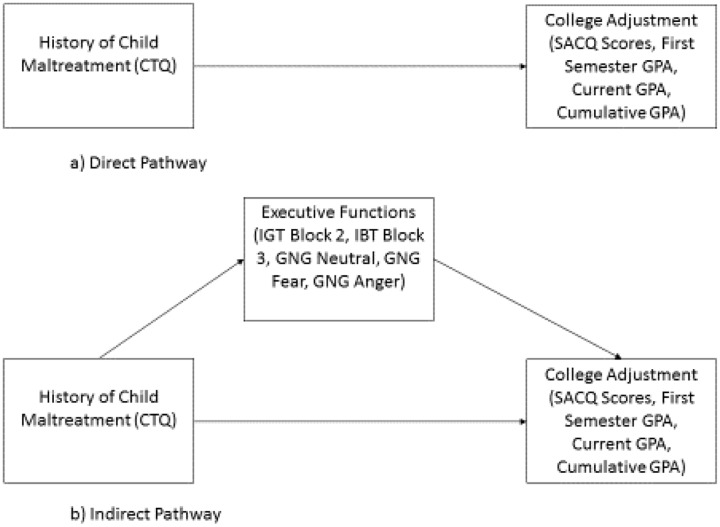
General mediation model tested in current study.

Based on the correlational patterns, GNG Neutral Face, Fear Face, and Anger Face RTs, as well as IGT Blocks 2 and 3 adaptive responding were tested as mediators of the relationship between the CTQ scores and academic measures (i.e., first semester GPA, cumulative GPA, GPA concurrent with testing semester, and total and subscale scores of the SACQ) using indirect effects analysis. See **Figure 1** for the general mediation model being tested.

**FIGURE 2:**
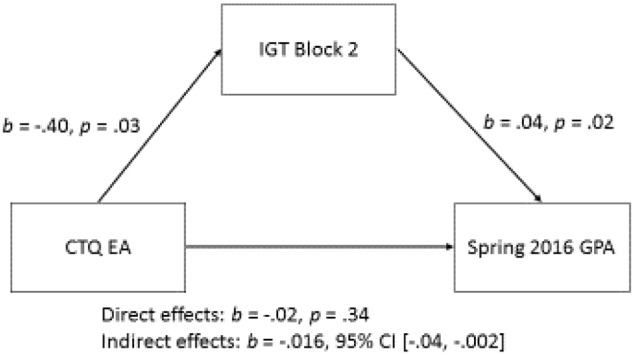
Path diagram for the significant indirect effect of IGT Block 2 on the association between CTQ Emotional Abuse and Spring 2016 GPA.

First, bivariate correlational analyses demonstrated that the CTQ Emotional Abuse (EA) and Total scores were marginally associated with concurrent and first-semester GPA, respectively (**Table 2**), thus, we examined the indirect effects of the potential mediators with the outcome of academic achievement. There was a significant indirect effect of CTQ Total Score on first semester GPA through IGT Block 2 adaptive responding, *b* = -0.005, BCa CI [-0.015, -0.0007]. This represents a small effect, η^2^ = 0.093, BCa CI [0.013, 0.231]. There was also an indirect effect of CTQ Emotional Abuse subscale score and Spring 2016 (concurrent with testing) GPA through IGT Block 2 adaptive responding, *b* = -0.02, BCa CI [-0.043, -0.002], which also represents a small effect, η^2^ = 0.094, BCa CI [0.01, 0.232] (see **Figure 2**).

**Table 2:** Direct effects of childhood maltreatment measures on college adaptation measures.

	First semester GPA	Spring 2016 GPA	Current Cumulative GPA	SACQ Academic	SACQ Social	SACQ Personal/ Emotion	SACQ Attachment	SACQ Total
CTQ Emotional Abuse	-0.15 (0.277)	-0.22 (0.09)	-0.16 (0.24)	-0.04 (0.73)	0.01 (0.94)	-0.18 (0.17)	0.15 (0.25)	-0.05 (0.68)
CTQ Physical Abuse	-0.15 (0.26)	-0.04 (0.75)	-0.08 (0.57)	0.12 (0.37)	0.10 (0.44)	0.08 (0.56)	0.07 (0.61)	0.12 (0.36)
CTQ Sexual Abuse	-0.17 (0.21)	-0.13 (0.32)	-0.18 (0.18)	-0.11 (0.39)	-0.17 (0.19)	-0.04 (0.74)	-0.12 (0.36)	-0.12 (0.35)
CTQ Emotional Neglect	-0.22 (0.09)	-0.16 (0.24)	-0.15 (0.26)	-0.22 (0.08)	-0.29 (0.02)*	-0.26 (0.05)*	-0.09 (0.47)	-0.28 (0.03)*
CTQ Physical Neglect	-0.13 (0.33)	-0.05 (0.7)	-0.09 (0.5)	-0.06 (0.62)	-0.13 (0.33)	-0.12 (0.35)	0.007 (0.96)	-0.09 (0.48)
CTQ Total	-0.23 (0.09)	-0.18 (0.19)	-0.19 (0.17)	-0.09 (0.49)	-0.12 (0.34)	-0.15 (0.24)	0.01 (0.92)	-0.12 (0.36)


**p*-values are included parenthetically; statistically significant correlations are denoted with ^∗^.*

Though correlations indicated that GNG Neutral Face RT, GNG Fear RT, and GNG Anger RT may serve as mediators through which the relationships between CTQ Total and CTQ Emotional Abuse and first semester GPA are effected, the indirect effects analyses did not support these potential mediators.

**FIGURE 3:**
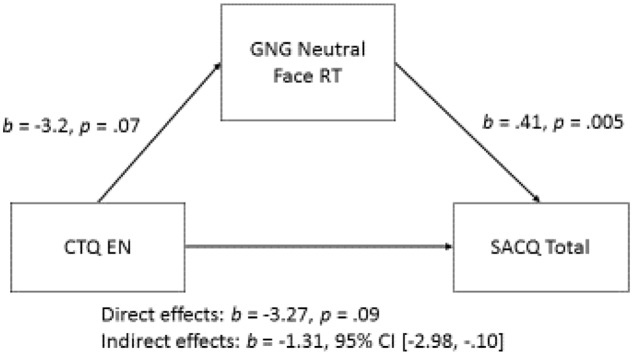
Path diagram for the significant indirect effect of GNG Neutral Face RT on the association between CTQ Emotional Neglect and Spring SACQ Total score.

Next, we examined the indirect effects of these mediators with the academic measure of total and subscale scores of the SACQ as the outcome variable. The bivariate correlational analyses demonstrated that the CTQ Emotional Neglect (EN) score was related to aspects of college adaptation (**Table 2**), and that GNG Neutral Face RT, and IGT Blocks 2 and 3 were potential mediators of this association. Regarding college adaptation, a consistent indirect effect of GNG Neutral Face RT on the relationship between CTQ Emotional Neglect and SACQ total (*b* = -1.314, BCa CI [-2.977, -0.102]), academic (*b* = -0.473, BCa CI [-1.138, -0.009]), social (*b* = -0.268, BCa CI [-0.809, -0.017]), and personal-emotional adaptation (*b* = -0.341, BCa CI [-0.829, -0.011]) scores was found. Measures of effect size for all indirect effects were small (η^2^ = 0.083, 0.073, 0.05, and 0.07, respectively) (see **Figure 3**).

Though correlations indicated that IGT Block 2 might mediate the relationship between CTQ EN and SACQ Total scores, the indirect effects analysis failed to support this by showing no effect of IGT Block 2 on this relationship. Indirect effects analysis also failed to support IGT Block 3 as a mediating variable in any analyses.

## Discussion

The purpose of this study was to add to a small, but emerging, literature regarding the extent to which a self-reported history of child-maltreatment (including emotional and physical abuse and neglect, and sexual abuse) predicts college outcomes in terms of GPA and self-reported adjustment (academic, social, etc.). While college students with trauma histories have been studied extensively, the focus has been mainly on mental health and other life adaptation outcomes, with surprisingly little attention paid to a critical milestone of emerging adulthood that has enormous public health value: success in college. We studied a volunteer sample of college undergraduates, as opposed to a sample of clinically diagnosed young adults (Radomski et al., 2016) or college students with documented PTSD symptoms (e.g., Kaysen et al., 2014). We believe this to be the first study to examine cognitive mediators between the self-reported maltreatment history and academic outcomes, in the form of hot and cool EF processes.

In our relatively heterogeneous volunteer sample we found that higher total scores on the CTQ, and on the Emotional Abuse and Neglect scales in particular, predicted poorer outcomes in terms of both GPA and self-reported adaptation. These findings are consistent with recent studies involving college students with a maltreatment history that have identified difficulties with adjustment to college (Banyard and Cantor, 2004; Elliot et al., 2009; Maples et al., 2014) and achievement as measured by GPA (Jordan et al., 2014), as well as increased risk for attrition (Duncan, 2000).

Our primary goal was to examine a mediational pathway from child maltreatment to college academic outcomes through EF, assessed in relatively cooler and warmer testing contexts. In light of the literature on EF deficits in children experiencing maltreatment, evidence that such deficits continue into young adulthood (Navalta et al., 2006), and documented deficits in adults with a trauma history (Navalta et al., 2006; Spann et al., 2012; Mothes et al., 2015; Vasilevski and Tucker, 2016), we wished to examine the indirect role of EF in predicting academic outcomes in this sample. We believe the evidence for impaired emotion regulation (Cloitre et al., 2005; Etkin and Wager, 2007) suggests that hot EF may be particularly vulnerable in this population. Aligned with these expectations, we identified small, but significant, indirect effects of EF processing in relatively hot conditions on the pathway between history of childhood maltreatment and academic outcomes in this sample of college students. Further, different hot EF tasks predicted different measures of academic success. This is the first published study that we are aware of to report indirect effects of EF processes between childhood maltreatment and adult college performance.

With regard to academic achievement, adaptive responding on the IGT mediated both the pathways between CTQ Total score and first-semester GPA, and CTQ Emotional Abuse score and GPA concurrent with testing. In the original IGT, a classic hot EF task, participants respond to feedback regarding gains and losses of hypothetical money in an arousing testing context. We further heated the task by providing a state lottery scratch ticket to participants who managed to complete the task with winnings, rather than an overall loss. During the second block of 20 IGT trials, we observed that individuals with higher scores on the CTQ (more reported overall maltreatment or specific emotional abuse) were less likely to shift their risky decision making to adaptive responses than individuals reporting lower levels of maltreatment. Our finding that deficits in hot EF in the form of IGT performance correlate with both reports of childhood emotional abuse and college GPA is a novel contribution to literature. It will be of interest to examine further how adaptive responding, reflecting learning from positive and negative feedback, is impacted by experiences of maltreatment during childhood, and may therefore impact success in academic settings.

With regard to self-reported adaptation to college, our findings suggest a different EF mediator, reaction time to Go stimuli in the Neutral Face Condition of the Go-No-Go Task of inhibition. This heated condition served as the significant indirect effect, rather than the cool Color Condition. We predicted that potential EF mediators would be found among our heated EF measures (IGT, Neutral and Emotion Face IGT reaction time and accuracy), and we did find evidence of this in the form of IGT adaptive responding and Go-No-Go Neutral Face reaction time. Individuals with higher scores on CTQ Emotional Neglect exhibited faster reaction times to the Neutral Face Condition of the Go-No-Go Task, and faster reaction times to this condition also predicted less positive adaptation to college in the academic, social, and personal–emotional domains. For a few reasons, we believe the use of faces as a heated stimulus makes good sense for our central research question. First, central importance of faces for social interaction is well appreciated. From early infancy, humans are “wired” to attend to faces and yet the development of face expertise continues into adulthood (Gliga and Csibra, 2007; Germine et al., 2011). In the maltreatment literature, many studies point to atypical face processing development (Pollak and Sinha, 2002). There is certainly evidence that development in a maltreatment milieu can be associated with relatively more exposure to negative facial expressions (Pollak and Sinha, 2002). Given the evidence for amygdala participation of processing all face emotions (though negative faces, in particular, Adolphs, 2002) and also just attention to faces in general (e.g., looking at the eye region of another, Adolphs, 2008), it also makes sense that individuals with maltreatment history may experience relatively greater arousal to faces. An important question raised by our results concerns the effect for neutral faces despite the lack of an effect for emotion face stimuli. First, we consider the neutral face stimuli that yielded an effect.

The notion that even neutral faces can serve as a potential threat stimulus such that face attention might yield individual differences in amygdala arousal was highlighted by a study by Schwartz et al. (2003). Following up on a longitudinal temperament investigation, this research team implemented a paradigm that involved viewing novel and familiar faces in an fMRI context. Performance was compared across two groups of young adults: one group had been classified as behaviorally inhibited at 2 years old (more likely to show fear to novelty) whereas the other group had been classified as behaviorally uninhibited (less likely to show fear to novelty). As young adults, the inhibited group showed greater amygdala activation to both familiar and novel faces with relatively stronger activation to novel faces. Thus, this study of normal individual differences in temperament makes clear that even neutral faces serve as an arousing for individuals with a more reactive amygdala.

We had originally hypothesized that the relatively hotter Emotion Face condition would yield relatively greater mediation (i.e., emotion greater than neutral). However, as demonstrated by the temperament study (reviewed above), it is a mistake to think of non-emotion faces as neutral stimuli. As powerful social stimuli, neutral faces are capable of generating arousal and they have been used effectively for eliciting individual differences in amygdala arousal. In our study design, all participants performed the neutral face block before the emotion face block. With this point in mind it is interesting to consider some additional alternatives. First, it is possible that for all participants the effect of the emotion content of the second block of faces was dampened by habituation. A second intriguing possibility is that for the purposes of our goal, non-emotion face stimuli had more power to discriminate between the groups. Following the literature, it is reasonable to hypothesize that all participants regardless of maltreatment history would show some arousal to threatening faces (e.g., Adolphs, 2002, 2008). Thus, it may be that the most sensitive stimuli for discriminating groups were the non-emotion faces. A third possibility that may be difficult to test concerns the lack of clarity among the findings using emotion faces with trauma groups. While there is evidence of atypical emotion face processing, there are conflicting findings as to which emotional displays (e.g., angry, fearful, happy) are more arousing and disruptive to performance (e.g., Caldwell et al., 2014; Cromheeke et al., 2014). Therefore, had we conducted separate conditions with a range of emotion faces (e.g., happy, fearful, angry, disgust), we may have observed specific indirect effects of a particular emotion face stimuli in this sample. It should be noted such a comprehensive emotion comparison would not be feasible in a single study such as ours, both because of the likely problem of habituation, discussed above, and because such a Go-No-Go paradigm with so many blocks would be excessive for a single participant.

Four particular limitations in our work deserve consideration. One limitation reflects difficulties inherent in the study of child maltreatment in an adult non-clinical sample. Following many other studies with college students we used a validated self-report measure, the CTQ, to examine individual differences in maltreatment history. We should emphasize that limitations associated with adult retrospective self-report of maltreatment status are limitations that challenge the broader field of researchers interested in exploring the impact of deleterious childhood events on adult adaptation. Adult self-report measures are likely to identify traumatic experiences among a larger percentage of the population of interest; however, it is also true that alternative measurement instruments (i.e., informant studies) are likely to miss cases (Stoltenborgh et al., 2014; Waxman et al., 2014). This issue may be especially clear when one considers emotional abuse in childhood, which is now recognized as a significant risk for adult outcome (e.g., Spertus et al., 2003). Unlike the kinds of child abuse that are likely to yield legal and medical evidence, emotional abuse may be unobserved and, therefore, require subjective participant report. We should note that when we administered a second instrument, the Trauma Symptom Checklist, both in our previous studies and in this present sample we found evidence that responses on the CTQ co-varied in predictable ways with trauma symptoms. Although, we did not seek out participants who were clinically diagnosed or in treatment (currently or in the past), we cannot determine the proportion of our volunteer sample that may fit this description. We assume that our sample is heterogeneous with respect to the specific profiles of trauma history (e.g., age and frequency of exposure), early risk and protective factors both at the levels of the individual (e.g., genetic vulnerability) and the context (e.g., neighborhood effects, etc.). Further, we assume risk and protective factors also vary in each individual’s current lives (e.g., possible adult trauma, other life stressors). It is important to stress that we cannot determine the degree to which our sample is biased based on our recruitment from an introductory psychology course participant pool. It is conceivable that students recruited from this course differ from other students at the university in ways we cannot account for. For example, introductory psychology courses may appeal more strongly to students who carry psychiatric or environmental risk. We are not aware of any college studies of maltreatment that satisfy methodological requirements to draw epidemiological conclusions. Our continued work in this area is designed to add more measures, such as clinical interviews, to better characterize our sample beyond the CTQ. The CTQ is a survey that is validated and well established in the literature, but still suffers from some degree of measurement error that could either depress or inflate correlations.

A second limitation is that our sample was majority female. To a large extent, this mirrors the demographics of the university population from which it was drawn; however, it may be the case that the title of our study selectively attracted female participants over males. It should be noted that a substantial proportion of studies examining history of childhood maltreatment involve exclusively female samples (e.g., Aspelmeier et al., 2007; Elliot et al., 2009; Kendra et al., 2012); nevertheless, our findings may not generalize to a more diverse population of individuals with maltreatment histories. To address this limitation, our future research will broaden our recruitment of participants. For example, we plan to include a sample of student veterans, a population that may be characterized by trauma at two developmental stages (Orcutt et al., 2003) and that is at high risk for academic difficulties and attrition (Rumann and Hamrick, 2007; DiRamio et al., 2008). The above limitations regarding the homogeneity of the sample highlight the potential problem of random versus non-random measurement error, likely inherent in most research in this domain. Non-random error causes underestimation of relationship, and presently we cannot disambiguate the degree to which our error reflects random or non-random factors. Recruitment of larger and more heterogeneous samples will allow for a more robust indirect effects mediational model to be identified with attention to potential model fitting errors.

A third limitation concerns our use of only two hot EF tasks in this study. It is important to note that we selected the two tasks carefully to be consistent with the relatively new and emerging hot EF literature. The IGT is considered to be a classic hot EF task as it is sensitive to the function of the ventromedial prefrontal cortex (Bechara et al., 1994; Damasio, 1999) and requires the execution of EF skills such as inhibition and flexibility under conditions of heightened arousal (i.e., incentives). The GNG is a well-established measure of inhibition that has been similarly “heated” with arousing stimuli (e.g., human faces) in past research (e.g., Hare et al., 2008), as we did in this study. Thus, we examined two accepted hot EF tasks, each with several indices of performance. We found that adaptive responding on a particular block of the IGT, and reaction time to the neutral face condition of the GNG were significant mediators between a history of maltreatment and GPA and self-reported college adaptation, respectively. Our future research will explore potential mechanisms (e.g., cognitive strategies, emotion regulation) underlying each of these findings. Further, we will continue to examine the degree to which other hot EF tasks, such as a heated working memory paradigm, demonstrate the same associations with both history of childhood maltreatment and college adaptation and achievement.

A fourth and final limitation involves our small effects. In this first study to examine the indirect effects of EF on the association between childhood maltreatment history and college outcomes, it is the case that the indirect effects we did identify were small, albeit significant. However, it should be noted that research designed to explain individual differences in the multifactorial domains of college adaptation and achievement (e.g., GPA) typically identify very small effects of single variables, even those factors much more proximal to the college outcomes (e.g., intelligence, self-efficacy, metacognition) than we examined in the current study (e.g., Murray and Wren, 2003; Kitsantas et al., 2008). Therefore, our small indirect effects utilizing only two hot EF tasks are very much in line with the effect sizes reported in the college adaptation and attrition literature, while also contributing new information regarding the potential pathways between history of maltreatment to college outcomes.

While we certainly acknowledge these various limitations, we must stress that this work represents the early stages of a novel approach to understanding an understudied subgroup of vulnerable college students. The overarching goal of our research program is to identify individuals within the large heterogeneous group of college students who might benefit from targeted interventions that are informed by our findings regarding the mediational pathways between childhood trauma and academic outcomes. From the start, our approach has rested on the assumption that any effort to identify patterns of relative risk within the large heterogeneous group with a maltreatment history must involve in exploration of current phenotype. The importance of understanding current cognitive and emotional factors reflects the multifactorial relationship between child developmental history and academic outcome. In this first, exploratory investigation we identified hot executive processes as a potential meditational connection. While our results should be considered preliminary and in need of further study, we believe that the examination of EFs within relatively hotter contexts may be a very fruitful direction for researchers interested in understanding the mechanisms that connect child maltreatment history to academic outcome. Promoting successful adaptation in the college setting may be one of our most effective means of contributing to positive life outcomes for individuals who carry developmental risk.

## Ethics Statement

This study was carried out in accordance with the recommendations of the Institutional Review Board for the Protection of Human Subjects in Research of the University of Northern Colorado, with written informed consent from all subjects. All subjects gave written informed consent in accordance with the American Psychological Association ethical guidelines. The protocol was approved by the Institutional Review Board of the University of Northern Colorado.

## Author Contributions

MW and EP collaborated in the conceptualization of the study, as well as in its design, execution, and the preparation of this manuscript. MJ contributed her expertise in data analysis and interpretation, as well as collaborated in the preparation of this manuscript.

## Conflict of Interest Statement

The authors declare that the research was conducted in the absence of any commercial or financial relationships that could be construed as a potential conflict of interest.

## Footnotes

1 http://www.macbrain.org/resources.htm
